# Physiological iodine uptake of the spine’s bone marrow in dual-energy computed tomography – using artificial intelligence to define reference values based on 678 CT examinations of 189 individuals

**DOI:** 10.3389/fendo.2023.1098898

**Published:** 2023-05-19

**Authors:** Philipp Fervers, Florian Fervers, Miriam Rinneburger, Mathilda Weisthoff, Jonathan Kottlors, Robert Reimer, David Zopfs, Erkan Celik, David Maintz, Nils Große-Hokamp, Thorsten Persigehl

**Affiliations:** ^1^ University Cologne, Faculty of Medicine and University Hospital Cologne, Department of Diagnostic and Interventional Radiology, Cologne, Germany; ^2^ Fraunhofer Institute of Optronics, System Technologies and Image Exploitation IOSB, Karlsruhe, Germany

**Keywords:** bone marrow, iodine, reference values, tomography, X-Ray computed, artificial Intelligence

## Abstract

**Purpose:**

The bone marrow’s iodine uptake in dual-energy CT (DECT) is elevated in malignant disease. We aimed to investigate the physiological range of bone marrow iodine uptake after intravenous contrast application, and examine its dependence on vBMD, iodine blood pool, patient age, and sex.

**Method:**

Retrospective analysis of oncological patients without evidence of metastatic disease. DECT examinations were performed on a spectral detector CT scanner in portal venous contrast phase. The thoracic and lumbar spine were segmented by a pre-trained neural network, obtaining volumetric iodine concentration data [mg/ml]. vBMD was assessed using a phantomless, CE-certified software [mg/cm3]. The iodine blood pool was estimated by ROI-based measurements in the great abdominal vessels. A multivariate regression model was fit with the dependent variable “median bone marrow iodine uptake”. Standardized regression coefficients (β) were calculated to assess the impact of each covariate.

**Results:**

678 consecutive DECT exams of 189 individuals (93 female, age 61.4 ± 16.0 years) were evaluated. AI-based segmentation provided volumetric data of 97.9% of the included vertebrae (n=11,286). The 95^th^ percentile of bone marrow iodine uptake, as a surrogate for the upper margin of the physiological distribution, ranged between 4.7-6.4 mg/ml. vBMD (p <0.001, mean β=0.50) and portal vein iodine blood pool (p <0.001, mean β=0.43) mediated the strongest impact. Based thereon, adjusted reference values were calculated.

**Conclusion:**

The bone marrow iodine uptake demonstrates a distinct profile depending on vBMD, iodine blood pool, patient age, and sex. This study is the first to provide the adjusted reference values.

## 1 Introduction

Neo-angiogenesis is a crucial molecular pathway to mediate malignant disease, since proliferating neoplastic cells require abundant supply with oxygen and nutrients (1). In dual-energy computed tomography (DECT), the elevated perfusion of a malignant tumor can be assessed by measuring the voxel-specific iodine concentration after intravenous contrast media application (2). By now, DECT has become a widely available technology and the iodine concentration is extensively obtained for the diagnosis, characterization, and treatment response monitoring of extra-skeletal malignant disease (3–10).

Despite its underrepresentation in recent literature, DECT iodine concentration imaging of the skeleton might be of particular interest, since conventional CT has only limited capability to detect malignant bone marrow disease (11, 12). Without dominating osteolytic or osteoblastic characteristics, bone marrow malignancy of the spine is regularly disguised by dense trabecular structure (12, 13). Compared to the gold standard magnetic resonance imaging (MRI) and positron emission tomography (PET), conventional CT only yielded a sensitivity of 0.77 and 0.63 to detect vertebral metastasis, or to diagnose bone marrow infiltration by Hodgkin lymphoma, respectively (11, 14). This diagnostic gap of conventional CT represents a major clinical limitation, since the spine is among the most common sites of malignant bone marrow infiltration (13–16).

To narrow this gap, several authors suggested to quantify the iodine concentration at small, non-specific lucencies or non-specific architectural distortions of the cancellous bone (17, 18). Further, the iodine concentration has been investigated to improve the diagnostic accuracy of the detection of spinal metastasis and to facilitate differential diagnosis of malignant spinal disease (6, 19–21). Despite its promising role in oncological skeletal imaging, there is a lack of reference values describing the normal iodine concentration of the spinal bone marrow in DECT. A possible explanation for this deficiency might be the expected dependence of vertebral iodine concentration assessment on the bone mineral density (BMD) and the iodine blood pool (22, 23).

The aim of this study was to provide an in-detail, adjusted database of the physiological iodine uptake of the spine’s bone marrow. Besides the dependence on the BMD and the iodine blood pool, we further aimed to investigate the influence of patient age and sex on the physiological bone marrow iodine uptake.

## 2 Materials and methods

All procedures performed in studies involving human participants were conducted in accordance with the ethical standards of the institutional (application number 21-1105) and national research committee and with the 1964 Helsinki declaration and its later amendments or comparable ethical standards. Informed consent was waived due to retrospective study characteristics.

Results are reported in line with the Strengthening the Reporting of Observational Studies in Epidemiology (STROBE) recommendations.

### 2.1 Patient enrollment

The patient population was enrolled by reviewing the institutional database for the below specified eligibility criteria.

#### 2.1.1 Inclusion criteria were:

1) Referral by the department of dermatology between May 2016 and January 2020 after resection of a malignant skin tumor to exclude metastatic disease,
2) DECT examination of the chest and abdomen using the below specified imaging protocol,
3) Patient age >18 years.

Follow-up examinations without evidence of macroscopic metastatic disease were included until January 2021.

#### 2.1.2 Exclusion criteria were:

1) Metal implants of the spine (n = 18 examinations of 5 patients),
2) Incomplete or corrupted DECT data (n = 26 examinations of 25 patients).

### 2.2 DECT imaging protocol

Patients were examined on a commercially available spectral detector DECT scanner (IQon Spectral CT, Philips Healthcare) in a head first, supine position. All scans were performed after intravenous administration of 100 ml iodine-based contrast agent with a flow rate of 3.0 ml/s (Accupaque 350, GE Healthcare). Contrast administration was followed by a saline flush of 30 ml. Bolus tracking in the descending thoracic aorta indicated portal venous phase with a delay of 50 s after surpassing a threshold of 150 HU. Tube voltage was 120 kV, the tube current was modulated by DoseRight 3D-DOM (Philips Healthcare). The collimation was 64 × 0.625 mm and the pitch 0.671.

### 2.3 DECT image reconstruction and post-processing

Anatomic conventional images were reconstructed using a hybrid-iterative reconstruction algorithm in “bone” preset (iDose4, convolution kernel C, Philips Healthcare). Spectral based raw data was processed to iodine concentration maps using the vendor’s proprietary software (IntelliSpace Portal 11.0, Spectral Diagnostics Suite, Philips Healthcare). All images were reconstructed in axial slices, 512 x 512 matrix, thickness 2 mm with an overlap of 1 mm. Voxel size was 0.89 x 0.89 x 2.00 mm.

### 2.4 Assessment of the covariate vBMD

Volumetric BMD (vBMD) was assessed phantomless by a CE-certified software in an standardized approach (IntelliSpace Portal 11.0, Bone Mineral Density, Philips Healthcare) (24). An ellipsoid volume of interest with a thickness of 9 mm was placed in the anterior portion of the vertebral body, sparing the surrounding cortical bone. Hence, vBMD measurements assessed only the cancellous part of the bone. In-body calibration to the mineral scale was achieved by analogous measurements in the paravertebral muscle and subcutaneous fatty tissue. To correct for the minor overestimation of vBMD measurements after intravenous contrast application, we applied the adjustment formula that was introduced by Abdullayev et al. and validated in consecutive research (6, 25–27). The correction formula is as follows: corrected vBMD = 0.88 * portal venous vBMD + 4.56 mg/cm3. Since the vBMD is a relatively stable biomarker (average expected change per year among women: 1.6%), we did not perform more than one measurement per patient and follow-up year (365 days) (28).

### 2.5 Assessment of the covariate iodine blood pool

The iodine blood pool was estimated by two region-of-interest-based, circular measurements in the portal vein and the abdominal aorta at the level of the kidney vessels, as suggested by previous studies (29, 30).

### 2.6 AI-based assessment of the bone marrow

**Figure 1:**
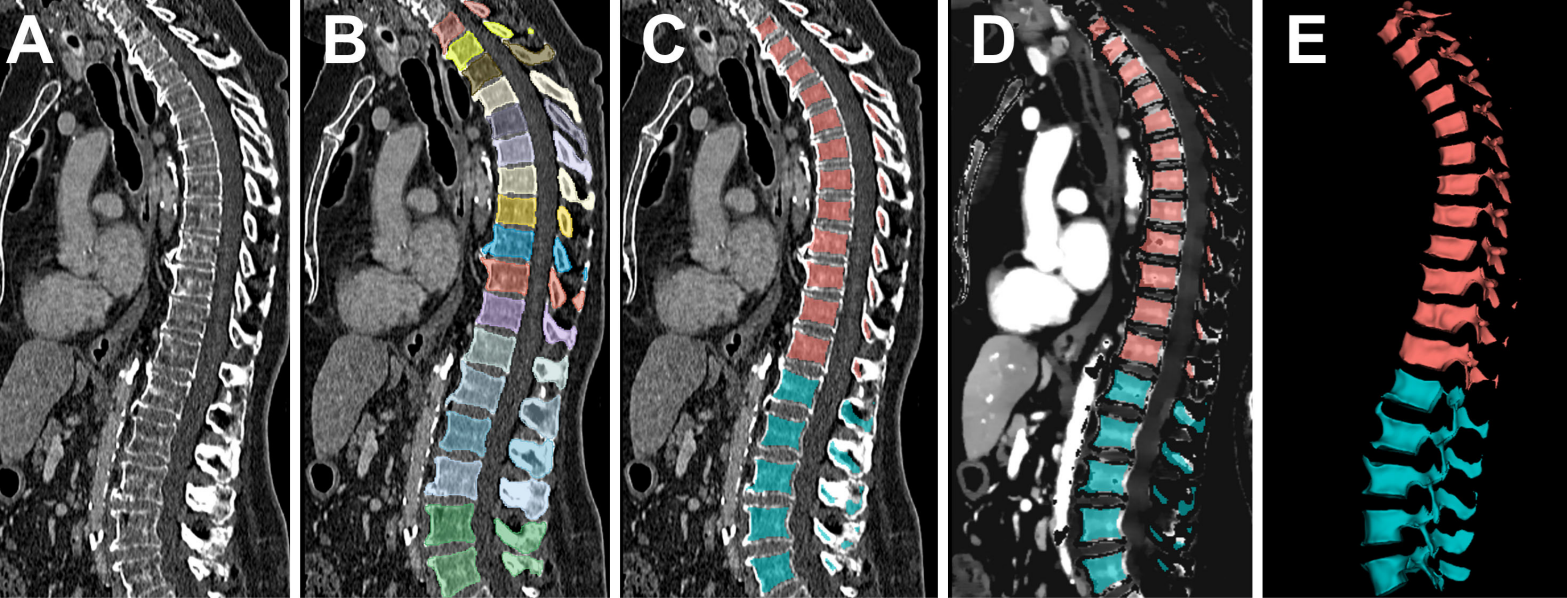
Automated assessment of the bone marrow iodine uptake. Conventional CT images served as the input to the pre-trained convolutional neural network by Payer et al. **(A)**. After automated, vertebra-by-vertebra segmentation of the spine **(B)**, the bottom-most five vertebrae were adopted as the lumbar segment, followed by the consecutive 12 vertebrae as the thoracic segment **(C)**. The segmentation was narrowed at each margin by 3 mm to exclude the bordering cortical bone, since it does not contain bone marrow. Consecutively, the bone marrow segmentation mask was transferred to the iodine concentration maps **(D)** and extracted as volumetric iodine concentration data for the lumbar and thoracic spine **(E)**.

The bone marrow was assessed by using artificial intelligence (AI), without requiring specific user interaction. First, the spine was segmented by a pre-trained, convolutional neural network by Payer et al. (31, 32). The neural network proved excellent performance with a dice coefficient of 0.94 and a correct vertebrae labelling rate of 0.99, based on analogous data to our study (120-kV acquisition with axial reformations, “bone” kernel favoring sharpness over noise, spatial resolution at least 1 mm) (33). After automated segmentation, the vertebrae were separated in lumbar and thoracic subsets. To exclude the bordering cortical bone, which does not contain bone marrow, the segmentation margins of each vertebra were narrowed by 3 mm using the SciPy command “scipy.ndimage.binary_erosion” (34). Consecutively, the thoracic and lumbar bone marrow segmentation masks were transferred from the conventional bone kernel image series to the iodine concentration maps. Automated assessment of the bone-marrow space is illustrated in **Figure 1**.

Evaluation of the iodine concentration of the bone marrow space

Voxel-wise iodine concentration was extracted from both the thoracic and lumbar bone marrow volumes as histograms ranging from -0.05 mg/ml to 19.95 mg/ml, with a bin size of 0.05 mg/ml.

### 2.7 Statistical data assessment

Statistical analysis was performed in *R* language for statistical computing, R Foundation, Vienna, Austria, version 4.0.0 (35). To test the data for normal distribution, we performed Shapiro-Wilk’s test using the *R* library *dplyr* (36).

To evaluate the dependence of the bone marrow iodine uptake on patient sex, age, vBMD, and iodine blood pool in the aorta as well as portal vein, a multivariate regression model was fit by *lm(y~x_1_+x_2_+x_3_+x_4_+x_5_)*, including the five above listed independent variables and the dependent variable “median bone marrow iodine concentration”. Both lumbar and thoracic iodine uptake were assessed in an individual model each. Multicollinearity was assessed by the variance inflation factor (VIF), calculated for each independent variable using the *R* library *cars* (a VIF <2.5 suggests no significant collinearity) (37, 38). In case of significant collinearity of one or more variables, those variables were excluded one at a time, starting with the highest VIF. After exclusion of multicollinearity, the remaining independent variables were ordered by their impact on the dependent variable, using the standardized regression coefficient β. Each independent variable’s β was calculated using the *R* library *QuantPsyc* (39). Finally, reference values of bone marrow iodine uptake were reported grouped by the independent variables, which mediated the strongest impact in the multivariate regression model (highest β). The power level of the final lumbar and thoracic regression models was calculated *post hoc* using the software G*Power for a sample size of 678 and a significance level of 0.05 (40).

Visualization was achieved using the *R* library *ggplot2* (41). Statistical significance was defined as p ≤ 0.05.

## 3 Results

In total, 678 DECT scans of 189 patients (93 female, patient age at examination 61.4 ± 16.0 years, range 24.2 – 90.8 years) were evaluated. The median number of included examinations per patient was 3 [2-5]. The most common type of resected malignancy was malignant melanoma (184/189, 97.3%), followed by Merkel cell carcinoma (3/189, 1.6%), and squamous cell carcinoma (2/189, 1.1%). Automated spine segmentation successfully yielded volumetric bone marrow data of 3390/3390 (100.0%) of included lumbar and 7896/8136 (97.1%) of included thoracic vertebrae, respectively. Mean number of included voxels was 95,987 ± 46,269 and 79,533 ± 50,127 per segmented lumbar and thoracic bone marrow space, respectively.

### 3.1 Assessment of the covariate vBMD

**Figure 2:**
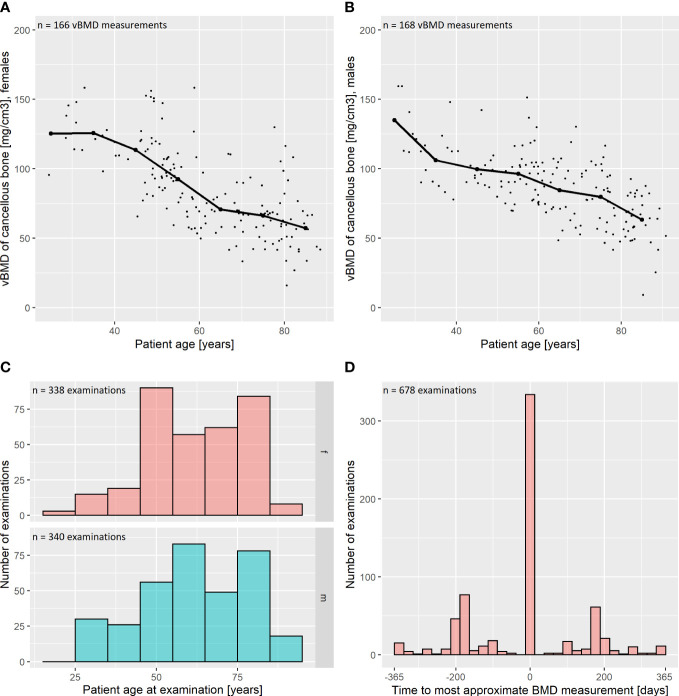
Descriptive statistics: bone mineral density and patient age. **(A, B)** Volumetric bone mineral density (vBMD) was assessed phantomless by an in-body calibrated, CE-certified software. The vBMD was corrected for intravenous contrast administration as suggested by Abdullayev et al., 2018 (25). Results are reported for female **(A)** and male **(B)** patients. The vBMD declines with increasing age of the assessed patients. Median values for intervals of 10 years are marked as thick scatter points with a connecting line. **(C)** Distribution of patient age in our study population, grouped by sex (f = female; m = male). **(D)** vBMD measurements were performed on 334 out of 678 included CT examinations (49.3%). For the remaining 344 examinations, the measurement of the most approximate CT was adopted for further analysis. In those cases, the mean time to the most approximate vBMD measurement was 192 ± 68 days, which is plotted as a histogram.

We performed vBMD measurements on 334/678 (49.3%) of the included DECT scans. For the other 344 examinations, which were not subject to vBMD quantification, the vBMD of the most approximate previous- or follow-up-scan was adopted for further analysis. In those cases, the mean time to the most approximate vBMD measurement was 192 ± 68 days. Mean vBMD was 85.6 ± 28.1 mg/cm3 (**Figure 2**).

### 3.2 Assessment of the covariate iodine blood pool

**Figure 3:**
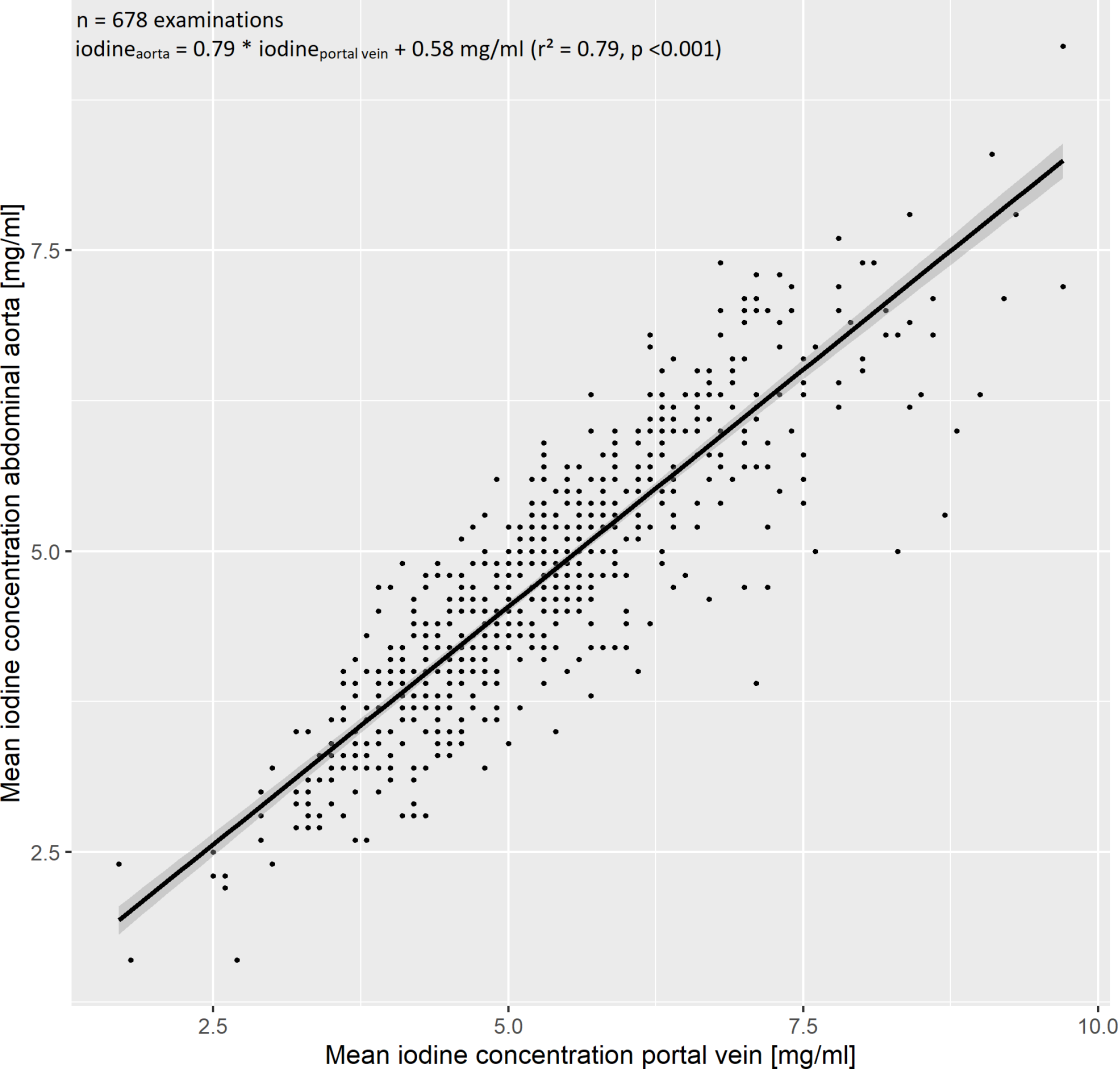
Evaluation of the iodine blood pool. The iodine blood pool was estimated by region of interest-based iodine concentration measurements in the abdominal aorta and portal vein. We observed a strong linear relationship, which was modelled by the regression equation iodine_aorta_ = 0.79 * iodine_portal vein_ + 0.58 mg/ml (r² = 0.79). The 95% confidence interval is marked by a grey band.

ROI-based measurements of the abdominal aorta and portal vein yielded a mean iodine concentration of 4.7 ± 1.1 mg/ml and 5.2 ± 1.2 mg/ml, respectively. We observed a strong, linear relationship of the median iodine concentrations in the aorta and portal vein, which was modelled by a linear regression with the formula iodine_aorta_ = 0.79 * iodine_portal vein_ + 0.59 mg/ml (r² = 0.78, **Figure 3**), indicating collinearity.

### 3.3 Fitting the multivariate regression analysis

**Figure 4:**
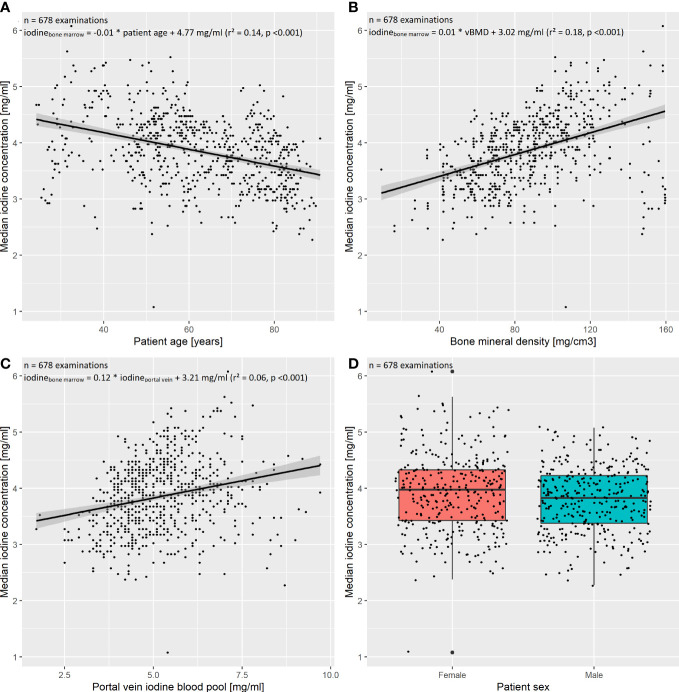
Multivariate regression of the thoracic spine’s bone marrow iodine uptake. The final multivariate regression model was fit including the independent variables **(A)** patient age, **(B)** bone mineral density (BMD), **(C)** portal vein iodine blood pool, as well as **(D)** patient sex, and the dependent variable “median bone marrow iodine concentration of the thoracic spine”. After exclusion of multicollinearity, the four independent variables remained significant regressors (p < 0.05). The multivariate model proved significant (F(4,673) = 89.53, p<0.001), with a multiple R²=0.34. The standardized regression coefficients β were calculated to order the independent variables by their impact on the dependent variable. The portal vein iodine blood pool had the largest impact on the thoracic bone marrow iodine uptake (β = 0.44), followed by the BMD, patient age, and sex (β = 0.35, β = -0.27, and β = 0.08, respectively).

To investigate the influence of patient age, sex, iodine blood pool (aorta as well as portal vein), and vBMD on the bone marrow’s iodine uptake, we fit two multivariate regression models with the five above enumerated independent variables and the dependent variable “median bone marrow iodine concentration of the lumbar spine” and “…of the thoracic spine”, respectively. The preliminary models including all five independent variables suggested collinearity of the iodine blood pool in the aorta and portal vein (VIF_aorta_ = 4.95, VIF_portal vein_ = 4.70). After exclusion of the abdominal aorta iodine blood pool, significant multicollinearity was ruled out (VIFs < 2.5). See **Figure 4** for the exemplary plot of the final thoracic model.

The final models are reported in-detail in **Table 1**.

**Table 1:** Final regression models to evaluate the relationships between the bone marrow iodine uptake and the included independent variables.

Model number	Localization	F, p, multiple R²	Power level	Independent variable	p	Standardized regression coefficient β
#1	Thoracic spine	F(4,673) = 89.53, p<0.001, R²=0.34	1.00	Patient age	<0.001	-0.27
				BMD	<0.001	0.35
				Portal vein iodine blood pool	<0.001	0.44
				Patient sex	0.03	0.08
#2	Lumbar spine	F(4,673) = 466.87, p<0.001, R²=0.74	1.00	Patient age	<0.001	-0.32
				BMD	<0.001	0.64
				Portal vein iodine blood pool	<0.001	0.41
				Patient sex	<0.001	0.17

The abdominal aorta iodine blood pool was excluded from both models due to extensive collinearity with the portal vein blood pool.

BMD, bone mineral density.

Within both final regression models, patient age, sex, vBMD, and the portal vein iodine blood pool were significant regressors. Mean standardized regression coefficients β were -0.30, 0.50, 0.43, and 0.13 for patient age, vBMD, iodine blood pool, and patient sex, respectively. I.e., assuming one standardized magnitude of change in iodine blood pool, a corresponding change of the vBMD had the 1.16-fold effect (0.50/0.43) on the bone marrow iodine uptake.

### 3.4 Quantitative features of the bone marrow’s iodine uptake

**Figure 5:**
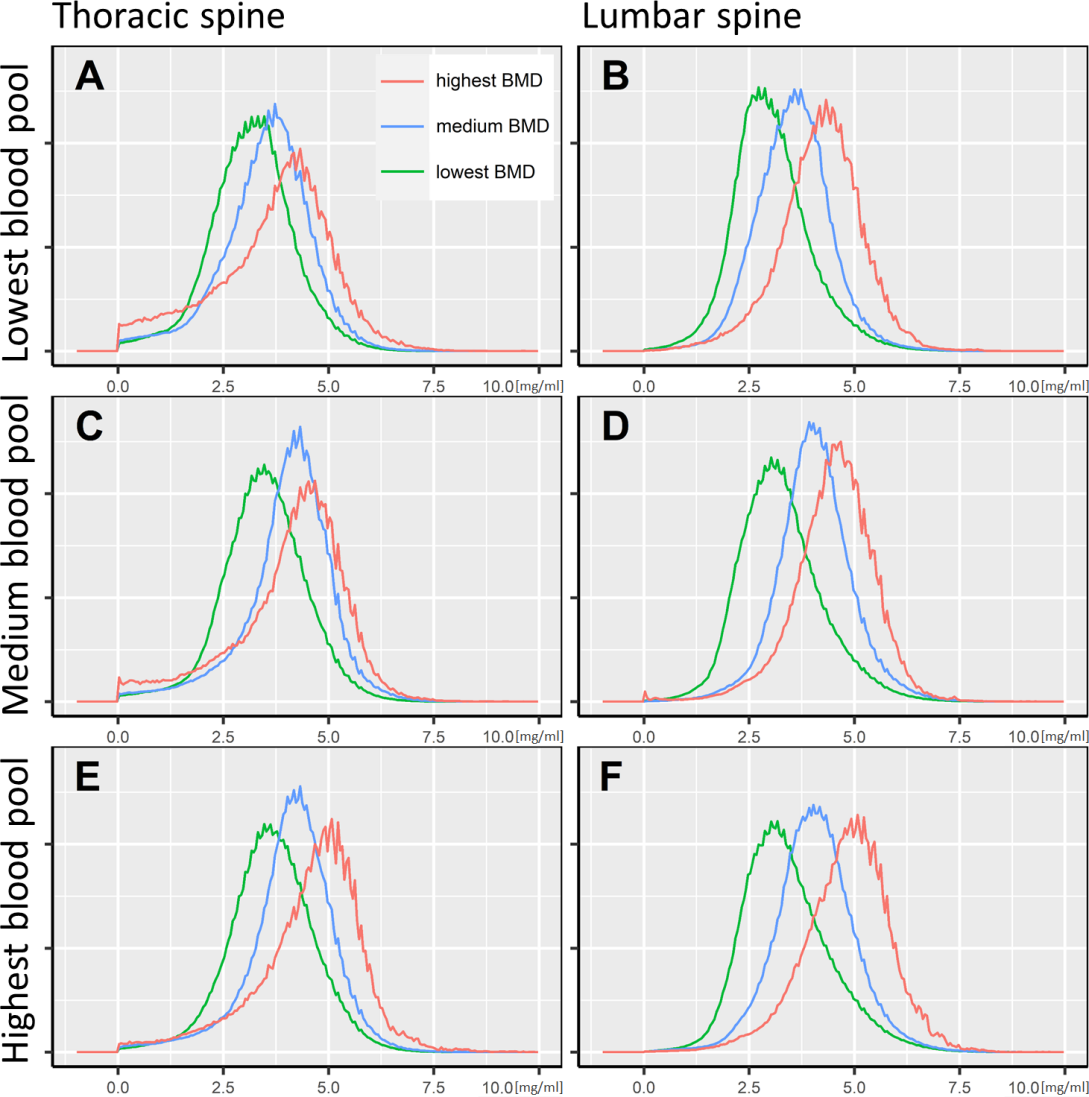
Bone marrow iodine uptake histograms. The iodine concentration distribution of the thoracic and lumbar spine’s bone marrow space is illustrated as histograms, standardized to a common spine volume. Accordingly, all histograms cover an identical area under the curve. The x-axis shows the voxel-wise iodine concentration in mg/ml. Six plots **(A–F)** are presented, grouped by the portal vein iodine blood pool and the bone mineral density. The cutoff for the lowest to highest iodine blood pool were <4.6 mg/ml, 4.6-5.6 mg/ml, and >5.6 mg/ml, respectively (A/B, C/D, and E/F). Regarding the bone mineral density, the cutoffs were <70.0 mg/cm3, 70.0-96.6 mg/cm3, and >96.6 mg/cm3, respectively (green, blue, and red lines).

Since the vBMD and the iodine blood pool mediated the strongest influence on the bone marrow’s iodine uptake, we report the quantitative iodine reference values grouped by those variables. The cutoff values to define the groups were determined by dividing the dataset into three equal parts, ordered by vBMD and iodine blood pool. **Figure 5** illustrates the results as histograms, while **Table 2** presents the in-detail descriptive statistics, including median, interquartile range, 95^th^ percentiles, and histogram maximum for each group. The equivalent data, yet grouped by patient age and sex, are documented in **Supplementary Material 1**.

**Table 2:** Descriptive statistics of the bone marrow’s iodine uptake, grouped by bone mineral density and the intensity of the iodine blood pool.

Intensity of iodine blood pool [mg/ml]	Bone mineral density [mg/cm3]	Thoracic spine				Lumbar Spine			
		Median	IQR	95^th^ perc.	Max.	Median	IQR	95^th^ perc.	Max.
<4.6	<76.0	3.2	2.6-3.8	4.8	3.3	3.0	2.4-3.6	4.7	2.7
	76.0-105.2	3.6	2.9-4.2	5.1	3.7	3.6	3.0-4.1	5.0	3.6
	>105.2	3.9	2.9-4.6	5.7	4.3	4.3	3.6-4.8	5.7	4.3
4.6-5.6	<76.0	3.5	2.9-4.1	5.1	3.5	3.2	2.6-3.8	5.1	3.0
	76.0-105.2	4.2	3.6-4.7	5.5	4.3	4.0	3.5-4.6	5.5	3.9
	>105.2	4.3	3.4-4.9	5.8	4.7	4.6	4.0-5.1	5.9	4.7
>5.6	<76.0	3.6	3.0-4.3	5.3	3.5	3.3	2.7-4.0	5.3	3.2
	76.0-105.2	4.2	3.6-4.7	5.6	4.3	4.1	3.5-4.6	5.6	4.0
	>105.2	4.7	3.9-5.3	6.2	5.1	4.9	4.2-5.5	6.4	5.1

Iodine concentration values are reported as median, quartiles, and 95th percentile, as well as the location of the maximum of the iodine concentration histogram.

IQR, Interquartile range; Perc., Percentile; Max., Maximum.

## 4 Discussion

Contrast enhanced CT is the most frequently performed imaging procedure for staging of solid cancer. Yet, there is a considerable chance to miss metastatic disease of the spine when examining a conventional CT scan with reported sensitivity of only about 0.77 (11, 12). To narrow this clinical limitation, several authors suggested to evaluate iodine concentration images from DECT, considering that malignant bone disease might stand out by elevated perfusion (6, 17, 18, 42). However, to date there is a lack of data about the physiological iodine uptake of the bone marrow, which is crucially required to delineate pathologically elevated iodine levels. The present study investigated physiological bone marrow iodine uptake on a sample of 11,286 vertebrae in 678 DECT scans, and presents the in-detail quantitative features as its main result. Further, we examined the influence of patient age, sex, the iodine blood pool, and the vBMD on the bone marrow iodine uptake.

The interdependent relation of bone mineral with iodine measurements is a well-studied observation, and might be a reason why the bone marrow iodine uptake is underrepresented among DECT studies. It is well-known, that a certain extent of misclassification of iodine and calcium in DECT material decomposition might occur due to similar x-ray absorption characteristics (6, 22, 26, 43). A recent phantom study at the identical DECT scanner as in our investigation reported a percentage error of iodine concentration in the presence of calcium of no more than 10% (<0.3 mg/ml) (22). Increasing the tube voltage from 120kV to 140kV in dual-layer CT might further reduce calcium-iodine misclassification, which could be exploited in dedicated bone marrow iodine uptake scans (6, 22).

In line with this inherent technical limitation, Borggrefe et al. reported that the iodine density in healthy trabecular bone depends on vBMD, which is, however, insignificant after inclusion of patient age (6). Contrarily, we found that the vBMD mediated the strongest impact on the bone marrow iodine concentration (p <0.001, mean β = 0.50). This finding might be promoted by inclusion of a relatively wide-spread age range of patients to our study (24-90 years), with a larger standard deviation of patient age compared to Borggrefe et al. (16.0 years vs. 12.7 years, respectively) (6). Particularly in young individuals with dense trabecular bone, iodine concentration assessment might be impaired, attributing a significant portion of the iodine concentration variance to vBMD. Yet, we consider the methodology of our study more appropriate to assess such multivariate effects: First, we evaluated a larger sample size (678 vs. 83 DECT examinations) with in total 11,286 vertebrae. Second, we avoided any subjective effects or inter-reader bias by relying on fully automated AI-segmentation of the bone marrow space. Last, the volumetric data in our study evaluated a by-magnitudes-larger iodine data pool than singular, ROI-based measurements. I.e., the mean number of included voxels per examination in our study was 175,520; assuming an in-plane resolution of 1 mm x 1 mm, a circular vertebral ROI with an exemplary diameter of 2 cm comprises only 314 voxels, implying the risk of sampling bias.

In line with Borggrefe et al., we also found a significant decrease of the bone marrow iodine uptake in elderly patients, which remained significant after adjusting for vBMD (p <0.001, mean β = -0.30). This age-dependent loss of bone marrow perfusion is a well-studied observation in histological studies and could be reproduced by our imaging results (44–46). Further, we investigated a significant impact of the iodine blood pool to the bone marrow iodine uptake (p <0.001, mean β = 0.43). This observation agrees with previous studies concerning parenchymatous abdominal organs (23, 29, 30).

Hence, we argue that the DECT bone marrow iodine concentration involves complex interactions, which should be recognized when adopting this imaging biomarker for clinical and scientific use – yet, there was no covariate that individually dominated our results. Assuming that iodine concentration reference values should be adjusted foremost to the vBMD and iodine blood pool, we report the quantitative results of our study accordingly, and provide a summary for clinical considerations in **Figure 5** and **Table 2**.

Recently, Borggrefe et al. examined iodine concentration thresholds to discriminate bone metastasis against healthy appearing trabecular bone on the identical dual-layer CT, using a similar scanning protocol as in our study (6). In their patient population (n=83 patients, mean age 64.6 years), the most performant cutoffs were investigated at an iodine concentration of 4.5-5.0 mg/ml, with a mean iodine concentration of bone metastasis of 5.6 ml/mg. In line with our investigation, Borggrefe et al. discuss that adjustment for vBMD and patient age might improve the performance of iodine concentration thresholds, yet they do not perform such analysis. Their investigated cutoffs locate close to the 95^th^ percentile of bone marrow iodine uptake in the respective age-group of our investigation, while the mean iodine uptake of bone metastasis surpasses the 95^th^ percentile (see **Supplementary Material 1**, respective 95^th^ percentile at 5.3 mg/ml). The definition of the 95^th^ percentile as an upper margin of the physiological range of a biomarker is a common approach, which enables clinical application of our results (13, 47–49). In clinical practice, an extraordinarily elevated iodine uptake above the adjusted 95^th^ percentile might hence favor the differential diagnosis of a malignant bone lesion.

This study has several limitations. First, the introduced reference values result from examinations using one specific scanner and imaging protocol. However, iodine concentration measurements have been proven robust between different DECT scanners of the latest generation, which supports generalizability of our findings (50–52). Concerning the warranted multi-vendor validation of our study, we assume our fully automated methodology excellently reproducible. The absence of specific user interaction allows for analysis of big data, which supports the warranted validation study. Although we evaluated a large-scale dataset of 678 examinations, patients at both extremes of the age spectrum are underrepresented. In particular the lack of younger patients is a frequent limitation of oncological research, since the age distribution of cancer prevalence is skewed towards the elderly (53, 54). Similar issues have been reported concerning the equity of racial and ethnic minorities in cancer research (55, 56). Hence, the ideal validation study of our data would comprise big data of multi-vendor, multi-center, multi-indication DECT with heterogenous age, sex, and ethnic distribution. To comply with the ethical standards, we did not perform DECT on healthy individuals without any medical history, but evaluated an oncological population. Albeit metastatic or recurrent disease was excluded, rare cases of undiagnosed malignancy cannot be ruled out. The same applies to possible systemic changes of physiology in oncological patients, e.g., blood pressure or kidney function, that might bias the bone marrow iodine uptake. Last, we did not include dual-energy X-ray absorptiometry (DEXA) as the gold standard to estimate the BMD. The vBMD was quantified in-body calibrated using a clinically approved vBMD tool, which is common practice in similar imaging studies and prevents for differences in BMD measurements between, e.g., the femur or radius at DEXA, compared to the spine in DECT (6, 24, 27, 57).

## 5 Conclusions

In conclusion, we present the first reference values of bone marrow iodine uptake in DECT, based on a large-scale cohort of 11,286 vertebrae in 678 examinations. Our multivariate analysis demonstrated that particularly the vBMD and the iodine blood pool affect the bone marrow iodine uptake, and hence should be adjusted for when adopting our results in clinical decision making. To achieve definite clinical evidence, a multi-center big-data study seems to be warranted by using the hereby provided AI-based workflow.

## Data availability statement

The raw data supporting the conclusions of this article will be made available by the authors, without undue reservation.

## Ethics statement

The studies involving human participants were reviewed and approved by Medizinische Fakultät der Universität zu Köln, Geschäftsstelle der Ethikkommission, Kerpener Str. 62, 50937 Köln. Written informed consent for participation was not required for this study in accordance with the national legislation and the institutional requirements.

## Author contributions

PF: Conceptualization, data curation, formal analysis, investigation, methodology, validation, visualization, writing – original draft, writing – review and editing. FF: Conceptualization, data curation, investigation, methodology, software, writing – review and editing. MR: Formal analysis, resources, visualization, writing – review and editing. MW: Formal analysis, resources, writing – review and editing. JK: Formal analysis, Resources, writing – original draft, writing – review and editing. RR: Data curation, investigation, writing – review and editing. DZ: Data curation, investigation, visualization, writing – review and editing. EC: Formal analysis, investigation, writing – review and editing. DM: Formal analysis, project administration, supervision, validation, writing – review and editing. NG-H: Conceptualization, project administration, supervision, validation, writing – review and editing. TP: Conceptualization, funding acquisition, project administration, supervision, validation, writing – original draft, writing – review and editing. All authors contributed to the article and approved the submitted version.
