# Supplementary material for: Physiological iodine uptake of the spine’s bone marrow in dual-energy computed tomography – using artificial intelligence to define reference values based on 678 CT examinations of 189 individuals
Source: Front Endocrinol (Lausanne). 2023 May 19;14:1098898. doi: 10.3389/fendo.2023.1098898 (PMC10235812; doi:10.3389/fendo.2023.1098898)
Supplement: Supplementary file 1 [file DataSheet_1.docx]

Quantitative features of bone marrow iodine uptake ordered by patient age and sex

Iodine concentration was extracted voxel-wise from the bone marrow space of the thoracic and lumbar spine. Median, 25^th^, 75^th^, and 95^th^ percentiles, ordered by patient age and sex, are reported for the thoracic and lumbar spine in Table 1. Further, the distribution of the iodine concentration is illustrated as histograms in Figure 1.

Table 1. In-detail description of the bone marrow iodine uptake.

| Localization | Patient age [years] | Female | | | | Male | | | |
| --- | --- | --- | --- | --- | --- | --- | --- | --- | --- |
|  |  | Median | IQR | 95^th^ perc. | Max. | Median | IQR | 95^th^ perc. | Max. |
| Thoracic spine | 20-39 | 4.7 | 3.8-5.4 | 6.5 | 5.0 | 3.9 | 2.9-4.7 | 5.8 | 4.3 |
|  | 40-59 | 4.2 | 3.4-4.8 | 5.7 | 4.3 | 4.0 | 3.2-4.6 | 5.5 | 4.2 |
|  | 60-79 | 3.7 | 3.1-4.4 | 5.3 | 3.8 | 3.8 | 3.1-4.5 | 5.5 | 4.0 |
|  | ≥80 | 3.4 | 2.8-4.1 | 5.1 | 3.3 | 3.5 | 2.8-4.1 | 5.2 | 3.3 |
| Lumbar  spine | 20-39 | 5.1 | 4.5-5.7 | 6.6 | 4.9 | 4.5 | 3.9-5.1 | 6.0 | 4.7 |
|  | 40-59 | 4.2 | 3.5-4.8 | 5.7 | 4.3 | 4.0 | 3.4-4.6 | 5.4 | 4.2 |
|  | 60-79 | 3.4 | 2.7-4.1 | 5.3 | 3.3 | 3.8 | 3.1-4.4 | 5.5 | 3.8 |
|  | ≥80 | 3.1 | 2.4-3.9 | 5.3 | 2.6 | 3.4 | 2.7-4.1 | 5.5 | 3.2 |

Iodine concentration values are reported in mg/ml. Besides median, quartiles, and 95^th^ percentile, the location of the maximum of the iodine concentration histogram is presented.
IQR = Interquartile range, Perc. = Percentile, Max. = Maximum


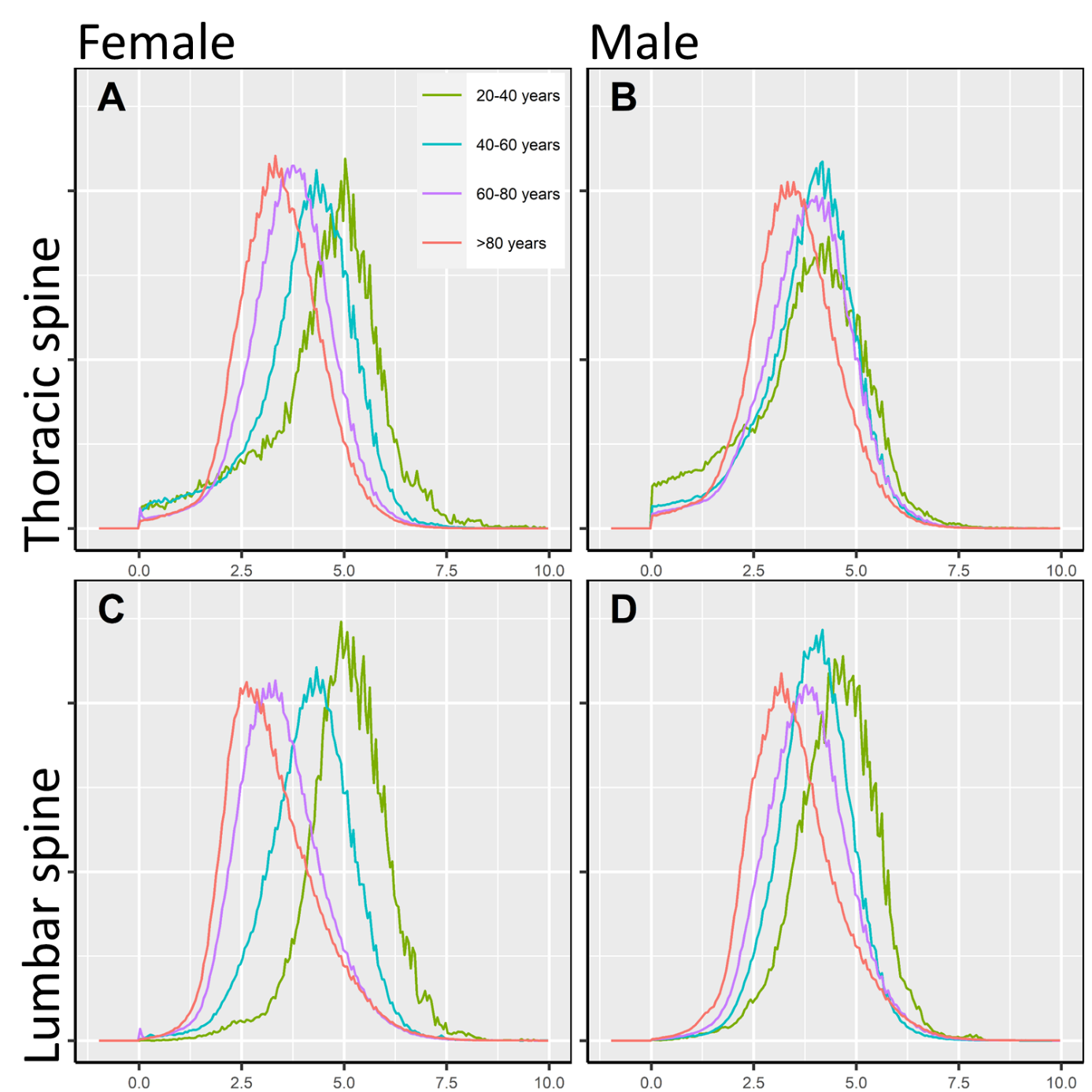


Figure 1. Distribution of the iodine concentration per patient age and sex.
The Iodine concentration (x-axis, mg/ml) was extracted voxel-wise from the three-dimensionally segmented bone marrow space. The results are illustrated as volume-standardized histograms grouped by patient age, sex, and the respective examined localization (thoracic/lumbar spine).
